# Exploring the Preventive and Therapeutic Mechanisms of Probiotics in Chronic Kidney Disease through the Gut–Kidney Axis

**DOI:** 10.1021/acs.jafc.4c00263

**Published:** 2024-04-04

**Authors:** Hsiao-Wen Huang, Ming-Ju Chen

**Affiliations:** † Department of Animal Science and Technology, National Taiwan University, No. 50, Ln. 155, Section 3, Keelung Road, Taipei 10673, Taiwan; ‡ Center for Biotechnology, National Taiwan University, No. 81, Changxing Street, Taipei 10672, Taiwan

**Keywords:** gut-dysbiosis, chronic kidney disease, gut−kidney axis, probiotic adjuvant therapy, host−microbe interactions

## Abstract

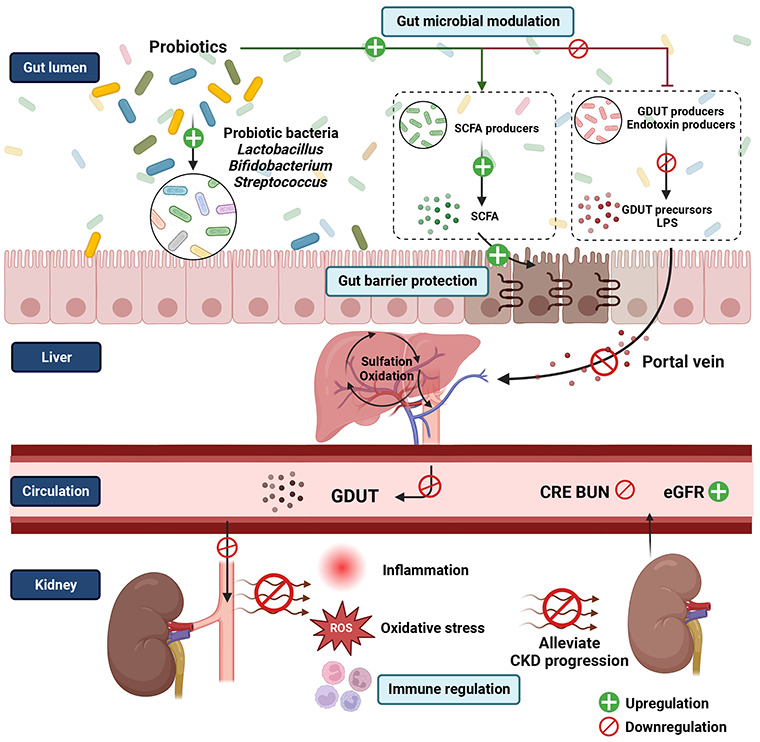

Gut dysbiosis contributes to deterioration of chronic kidney disease (CKD). Probiotics are a potential approach to modulate gut microbiota and gut-derived metabolites to alleviate CKD progression. We aim to provide a comprehensive view of CKD-related gut dysbiosis and a critical perspective on probiotic function in CKD. First, this review addresses gut microbial alterations during CKD progression and the adverse effects associated with the changes in gut-derived metabolites. Second, we conduct a thorough examination of the latest clinical trials involving probiotic intervention to unravel critical pathways via the gut–kidney axis. Finally, we propose our viewpoints on limitations, further considerations, and future research prospects of probiotic adjuvant therapy in alleviating CKD progression. Enhancing our understanding of host–microbe interactions is crucial for gaining precise insights into the mechanisms through which probiotics exert their effects and identifying factors that influence the effectiveness of probiotics in developing strategies to optimize their use and enhance clinical outcomes.

## 1 Introduction

Chronic kidney disease (CKD) is characterized by a substantial loss of kidney function and is associated with consistent exposure to numerous risk factors such as hypertension, diabetes mellitus, and obesity, leading to the irreversible progressive decline of kidney excretory function. The accumulation of uremic toxins in the circulation that are normally excreted by healthy kidneys causes massive damage to the kidney. Typically, kidney fibrosis is involved in CKD development, leading to myofibroblast activation, migration, accumulation of extracellular matrix, and kidney failure.^1^ CKD also impacts other organs and tissues, especially the cardiovascular system. Left ventricular hypertrophy is highly prevalent in patients with CKD, which is strongly associated with systolic hypertension and eventually results in heart failure, a leading cause of morbidity and mortality in this population.^2,3^

The CKD diagnosis is based on kidney function and structure abnormalities that persist for >3 consecutive months. The Kidney Disease Improving Global Outcomes (KDIGO) classifies CKD based on the estimated glomerular filtration rate (eGFR) into five stages: >90 mL/min per 1.73 m^2^ (stage 1), 60–89 mL/min per 1.73 m^2^ (stage 2), 30–59 mL/min per 1.73 m^2^ (stage 3), 15–29 mL/min per 1.73 m^2^ (stage 4), and <15 mL/min per 1.73 m^2^ (stage 5). The extent of albuminuria is also an additional indicator of end-stage renal disease (ESRD) progression.^4^

The global CKD prevalence is increasing, affecting over 10% of the global population and accounting for over 843.6 million individuals worldwide.^5^ Unfortunately, there is no cure for CKD, with treatments, including lifestyle changes, medication, and dialysis, only assisting in relieving symptoms and delaying their progression. A kidney transplant may also be a treatment option for patients suffering from ESRD. However, the limited number of donated kidneys and lengthy waiting periods impede its accessibility. Therefore, CKD is becoming a severe public health issue, requiring a better solution to ameliorate and alleviate its progression.^6^

Accumulating clinical evidence supports the theory that gut dysbiosis significantly contributes to deteriorating CKD progression, generating gut-derived uremic toxin (GDUT) and aggravating kidney failure.^7−9^ Therefore, strategies based on microbiota-based interventions could be considered preventive and therapeutic approaches to modulate gut microbiota and their metabolites to alleviate CKD progression. Probiotics are “live microorganisms that, when administered in adequate amounts, exist a health benefit on the host”.^10^ They are well-recognized for modulating gut microbiota, and research is ongoing regarding their efficacy in preventing and managing CKD. The efficacy of probiotics in decreasing uremic toxin production and improving renal function has been investigated using *in vitro* models^11,12^ and various animal^11,13^ and human CKD studies.^14^

To provide a critical perspective on the potential of probiotics as preventive and therapeutic approaches in CKD, we first systematically delve into gut dysbiosis and its interplay with gut microbiota, associated metabolites, and CKD progression. Subsequently, we evaluate the latest studies examining the efficacy of probiotics in clinical cohorts and comprehensively review their mechanisms of action in CKD through the gut–kidney axis. Finally, we discuss the limitations, further considerations, and future research prospects regarding the utilization of probiotics as adjuvant therapy to enhance outcomes in patients with CKD.

## 2 Strong Link between CKD and Gut Microbial Dysbiosis

The intestinal microbiota is markedly altered in CKD due to multifactorial causes, including iatrogenic effects and altered physiological conditions.^15,16^ Antibiotics, phosphate binders, dietary restriction, and low fiber consumption lead to slow intestinal transit, impaired protein assimilation, metabolic acidosis, and constipation.^17^ CKD-induced adaptive secretion of uric acid by the colon also limits the increase in serum uric acid concentration.^18^ More undigested protein and urea/uric acid in the colon enrich the proliferation of proteolytic bacteria with urease, uricase, phenol-, and indole-forming enzymes, whereas the short-chain fatty acid-producing bacteria are decreased.^9^ The increased intestinal pH through the augmented secretion of urea into the gut, along with limited carbohydrate fermentation, favors disruption of the intestinal epithelial barrier, and the production of phenolic and indolic compounds by colonic bacteria.^19^ This CKD-induced gut dysbiosis increases the production of GDUT and decreases the level of short-chain fatty acid (SCFA) such as butyric acid. The alteration of the gut environment affects the gut barrier by disrupting the colonic epithelial tight junctions, which in turn facilitates the movement of endotoxin, pathogenic microbes, and bacterial fragments into the systemic circulation that triggers inflammation.^20^

Additionally, the bladder microbiotas also plays a pivotal role in urinary tract infections, interstitial cystitis, urinary incontinence, and kidney stones.^21^ To date, the literature discussing the relationship between gut microbiota and bladder microbiota is limited. However, kidney function undoubtedly impacts the bladder microbiota, as evidenced by Hrbacek et al. (2022), who found distinct overall microbial compositions between patients with CKD and healthy individuals.^22^ Furthermore, Kramer et al. (2018) discovered that among patients with CKD, those with better kidney function (higher eGFR) exhibited greater diversity in their urinary microbiota.^23^ The composition of a patient’s urine, which affects the urinary microbiota, can be influenced by kidney function. For instance, a significant decrease in eGFR leads to reduced concentrations of uromodulin (also known as Tamm–Horsfall glycoprotein), produced by renal tubules. Because uromodulin has bacteriostatic properties, its diminished presence affects bacterial growth.^24^ Therefore, CKD affects the diversity and composition of the bladder/urinary microbiota, subsequently influencing urinary tract symptoms and bladder health.

### 2.1 Gut-Derived Uremic Toxins Play a Significant Role in CKD Progression

Uremic retention solutes, so-called uremic toxins, are normally excreted by the healthy kidney but accumulate in patients with CKD and contribute to biological dysfunction.^25^ They can be categorized into three major types: (1) free water-soluble molecules with a molecular weight <500 Da that readily pass the dialysis filter with less toxicity (e.g., trimethylamine, urea, oxalate, creatinine); (2) middle molecules with a molecular weight ≥500 Da that have limited passing capacity due to the dialysis membrane characteristics (e.g., adiponectin, cystatin C, TNF-α); (3) low molecular weight protein-bound molecules which their dialytic removal predominantly depends on the equilibrium between bound and free molecules (e.g., indoxyl sulfate, *p*-cresyl sulfate) (Table 1).^26^ Uremic toxins can also be classified according to the site of origin,^27^ such as GDUT, produced from microbial metabolism. Gut microorganisms can deaminate or decarboxylate amino acids.^28^ Deamination of aromatic amino acids (tyrosine, tryptophan, and phenylalanine) leads to the formation of phenolic compounds (indole, *p*-cresol, and phenol). Consequently, gut microbial dysbiosis could increase the production of GDUT precursors and generate more uremic toxins.^29^

**Table 1:** Classification of Uremic Toxins^a^,^25,37^

categories	numbers	MW	molecules
free water-soluble molecules	45	<500 Da	• creatinine
			• hypoxanthine
			• oxalate
			• SDMA
			• TMA/TMAO
			• urea
			• uric acid

middle molecules	22	≥500 Da	• adiponectin
			• cystatin C
			• cytokine: IL-6, IL-8, IL-10, and TNF-α
			• fibroblast growth factor-23
			• glutathione (oxidized)
			• leptin
			• retinol-binding protein
			• VEGF

protein-bound molecules	25	<500 Da (most of them except for leptin and retinol-binding protein)	• 4-ethylphenyl sulfate
			• CMPF
			• hippuric acid
			• indoleacetic acid
			• indoxyl glucuronide
			• indoxyl sulfate
			• kynurenic acid
			• leptin
			• *p*-cresol
			• *p*-cresyl glucuronide
			• *p*-cresyl sulfate
			• phenol
			• phenyl glucuronide
			• phenyl sulfate
			• phenylacetic acid
			• phenylacetyl glutamine
			• retinol-binding protein

total	90	leptin and retinol-binding protein exhibit distinctive characteristics in two groups	

a Da, Dalton; SDMA, symmetric dimethylarginine; TMA, trimethylamine; TMAO, trimethylamine-*N*-oxide; IL, interleukin; TNF-α, tumor necrosis factor α; CMPF, 3-carboxy-4-methyl-5-propyl-2-furanpropionic acid; VEGF, vascular endothelial growth factor.

GDUTs have recently gained attention for their role in CKD as they cause detrimental effects on renal, vascular, cardiac, and other tissues and organs.^30^ Currently, five GDUTs are associated with cardiovascular disease and mortality in CKD as well as other end-organ toxicity: indoxyl sulfate (IS), indole-3 acetic acid (IAA), *p*-cresyl sulfate (PCS), phenylacetylglutamine (PAG), and trimethylamine-*N*-oxide (TMAO).^31−33^ Dietary protein is the source of IS, IAA, PCS, and PAG, whereas IS and IAA are protein-bound uremic toxins generated from dietary tryptophan. Indole, a precursor of IS, is a product of tryptophan produced by intestinal microorganisms, which is converted into an IS through sulfation in the liver after intestinal absorption. IAA biosynthesis is linked to the metabolism of tryptophan and involves the elimination of amino and carboxyl groups from tryptophan α-carbon via intermediates such as indolepyruvate, indoleacetamide, or indoleacetonitrile.^34^ PCS and PAG are derived from tyrosine and phenylalanine, which are metabolized and converted into *p*-cresol by intestinal proteolytic microorganisms in the colon, then absorbed into the circulation and subsequently undergo oxidization and sulfation by the liver to produce PCS.^35^ PAG biosynthesis is also involved in phenylalanine metabolism. Gut microbiota fermented unabsorbed phenylalanine to produce phenylacetate and conjugate with glutamine to generate PAG in the liver.^36^ Trimethylamine (TMA), a precursor of TMAO, is produced by bacterial metabolism of quaternary amines such as choline, phosphatidylcholine, betaine, and l-carnitine. TMA is subsequently absorbed and oxidized by the hepatic enzyme flavin monooxygenase isoform 3 (FMO3) to form TMAO in the liver.^3^

IS, IAA, PCS, and PAG are the protein-bound molecules with high binding affinity for albumin.^37^ These albumin complexes are transported to proximal renal tubular cells and excreted via organic anion transporter (OAT) 1 and OAT3, located on the basolateral membrane of tubular cells.^38^ OATs are inhibited by high levels of GDUTs as kidney function declines.^39^ Accumulation of IS, IAA, PCS, and PAG in circulation is toxic due to the limited dialytic clearance by conventional hemodialysis and their adverse health impact, resulting in cardiovascular complications and mortality in patients with CKD.^40^ Additionally, IS and PCS increase oxidative stress in renal tubular cells associated with CKD progression and its comorbidities.^41^ TMAO is efficiently removed by dialysis, but it is a risk factor for CKD and cardiovascular disease (CVD).^42^ A clinical trial of 4007 patients concluded that elevated plasma TMAO levels are associated with an increased risk of incident major adverse cardiovascular events such as death, myocardial infarction, or stroke during three years of follow-up.^43^

### 2.2 Gut Microbial Alternation in Patients with CKD is a Potential Biomarker for the Detection of Kidney Disease

Numerous studies have reported CKD-associated changes in the human gut microbiome composition, indicating strong links between CKD and gut microbial dysbiosis (Table 2). Based on previous studies with more than 75% consistent findings, patients with kidney disease have a higher relative abundance of *Proteobacteria*, *Enterobacteriaceae*, *Streptococcaceae*, *Streptococcus*, *Bilophila*, *Desulfovibrio*, *Klebsiella*, *Escherichia-Shigella*, and lower abundance of *Firmicutes*, *Prevotellaceae*, *Prevotella*, *Prevotella* 9, *Alcaligenaceae*, *Roseburia*, *Faecalibacterium*, and *Faecalibacterium prausnitzii* in comparison to healthy subjects.^44^ Immunoglobulin A (IgA) nephropathy patients have increased *Ruminococcaceae*, *Lachnospiraceae*, *Eubacteriaceae*, and *Streptococcaeae* in *Firmicutes,* while *Bifidobacterium* and species of *Clostridium*, *Enterococcus*, and *Lactobacillus* were decreased compared to healthy controls.^45^

**Table 2:** Summary of the Current Literature Supporting Gut Dysbiosis in Patients with Chronic Kidney Disease^a^

subjects	alteration of gut microbiota	main findings	ref
immunoglobulin (Ig) A nephropathy patients	• elevation of some genera/species of *Ruminococcaceae*, *Lachnospiraceae*, *Eubacteriaceae*, *Streptococcaeae*, *Sutterellaceae*, and *Enterobacteriaceae*	• IgAN patients had an altered fecal microbiota	(45)
	• reduction of *Bifidobacterium and* species of *Clostridium*, *Enterococcus and Lactobacillus*		

HD patients	• elevation of enterobacteria (*Klebsiella* and *Escherichia coli*), enterococci, and *Clostridium perfringens*	• an overgrowth of aerobes of the fecal microbiota in HD patients is responsible for a high accumulation of *p*-cresol and indole	(81)
	• reduction of bifidobacteria		

CKD stage 2–3 patients	• elevation of the phyla *Actinobacteria* and *Proteobacteria*	• lower intestinal flora diversity and abundances	(49)
	• elevation of the genera *Bacteroides*, *Escherichia*, *Ruminococcus*, *Blautia*, *Enterococcus*, *Clostridium*, *Eubacterium*, *Klebsiella*, *Sarcina*, *Eggerthella*, *Turicibacter*, *Bilophila* and *Pseudoramibacter*	• *Ruminococcus* and *Roseburia* displayed the highest diagnostic values for distinguishing CKD patients from healthy controls	
	• reduction of the genera *Roseburia*, *Prevotella*, *Faecalibacterium*, *Megamonas*, *Coprococcus*, *Burkholderia*, *Dialister*, *Lachnospira, Streptococcus*, *Megasphaera*, *Sutterella, Collinsella*, *Stenotrophomonas*, *Haemophilus*, *Odoribacter*, *Butyricimonas*, *Acidaminococcus*, and *Granulicatella*		

ESRD patients	• elevation of *Bacteroides*, *Escherichia*/*Shigella*, *Subdoligranulum*, *Fusobacterium*	• this reduction in beneficial bacteria may play an important role in the pathogenic processes of CKD	(106)
	• reduction of *Prevotella, Roseburia, Faecalibacterium, Clostridium, Coprococcus, Dorea.*		

HD patients and nondialyzed CKD patients	• elevation of the phyla *Bacteroidetes and Proteobacteria*	• the differential gut microbiota has the potential to guide noninvasive diagnosis and targeted interventions	(119)
	• elevation of the genera *Bacteroides*, *Escherichia Shigella*, *Parabacteroides*, *Ruminococcus gnavus* group, *Ruminococcus torques* group, *Weissella*, *Flavonifractor*, *Ruminiclostridium 5*, *Sellimonas*, *Erysipelatoclostridium*, *Eggerthella*, and *Clostridium innocuum* group	• *Holdemanella*, *Megamonas*, and *Prevotella2* were exhibited the highest abundance, whereas *Dielma* and *Scardovia* were absent in healthy controls; these genera could be an indicator of the progression of CKD and HD	
	• reduction of the phyla *Firmicutes*		
	• reduction of the genera *Dialister*, *Eubacterium rectale* group, *Carnobacterium, Lachnospira*, *Subdoligranulum*, *Eubacterium coprostanoligenes* group, *Coprococcus 2*, *Roseburia*, *RuminococcaceaeUCG 009, Ruminococcaceae NK4A214* group, *Lachnospiraceae FCS020* group, *Ruminococcus1*, *Romboutsia*, *Butyricicoccus*, *Collinsella*, *RuminococcaceaeUCG 003*, *Eubacterium hallii* group, *Tyzzerella3*, and *LachnospiraceaeUCG 001*		

ESRD patients	• elevation of the families *Enterobacteriaceae*, *Halomonadaceae*, *Moraxellaceae*, *Polyangiaceae*, *Pseudomonadaceae*, and genera *Brachybacterium*, *Catenibacterium*, *Nesterenkonia*, and *Thiothrix* were markedly increased in patients with ESRD	• ESRD significantly modifies the composition of gut microbiome in humans; uremia and the strict dietary restrictions must have contributed to the observed changes in their microbial flora	(111)
	• reduction of the families *Sutterellaceae*, *Bacteroidaceae*, *and Lactobacillaceae*		

CKD and ESRD patients	• at the phylum level, the relative abundance of *Proteobacteria* was enriched in the ESRD group, whereas *Euryarchaeota* was more abundant in the healthy control group	• a decrease in 14 SCFA-producing bacteria in the CKD group, including *R. bromii*, *R. callidus*, *R. hominis*, *E. rectale*, *F. prausnitzii*, *C. comes*, *C. eutactus*, *C. sporogenes*, *S. variabile*, *D. succinatiphilus*, *B. adolescentis*, *L. crispatus*, *A. indistinctus*, and *A. inops*, which could promote CKD progression by impairing intestinal barrier function and stimulating excessive inflammation	(7)
	• at the family level, *Veillonellaceae*, *Lactobacillaceae*, and *Eubacteriaceae* significantly decreased, but *Enterobacteriaceae* gradually increased in the ESRD group. *Rikenellaceae*, *Lactobacillaceae*, and *Clostridiaceae* decreased in the moderate CKD group, and *Selenomonadaceae* increased in the mild CKD group	• the distinct changes in gut microbiota were associated with alterations in the metabolic functions of arginine and proline, arachidonic acid, and glutathione, as well as in the biosynthesis pathways of ubiquinone and other terpenoid-quinone compounds during CKD progression	
	• at the genus level, *Roseburia*, *Faecalibacterium*, *Eubacterium rectale*, *Eubacterium*, and *Ruminococcus* considerably decreased in the moderate CKD and ESRD groups, whereas *Flavonifractor* and *Citrobacter* increased in these groups	• the disrupted microbiota in relation to CKD severity may be implicated in an imbalanced toxic and pro-oxidant metabolism within both the gut and host, ultimately accelerating the CKD progression, which may represent a valuable early diagnostic and therapeutic target for CKD	
	• Species varied across CKD progress:		
	Four species increased (*Citrobacter freundii*, *Citrobacter werkmanii*, *Flavonifractor plautii*, and *Anaerostipes caccae*) during CKD progression		
	Fourteen species decreased (*Methanobrevibacter smithii*, *Coprococcus comes*, *Coprococcus eutactus*, *Clostridium sporogenes*, *Ruminococcus callidus*, *Ruminococcus bromii*, *Roseburia hominis*, *Faecalibacterium prausnitzii*, *Veillonella parvula*, *Megasphaera elsdenii*, *Dialister succinatiphilus*, *Acidaminococcus intestini*, *Faecalicoccus pleomorphus*, and *Subdoligranulum variabile*) during CKD progression		
	• Species varied in only a specific CKD group:		
	*Megasphaera micronuciformis* level increased in the mild CKD group		
	*Alistipes indistinctus*, *Alistipes inops*, and *Bacteroides uniformis* levels decreased in the moderate CKD group		
	*Turicibacter sanguinis* level increased, but the *Streptococcus mutans*, *Bifidobacterium adolescentis*, and *Lactobacillus crispatus* levels decreased in the ESRD group		

ESRD patients	• the most enriched species in patients included *Eggerthella lenta*, *Flavonifractor* spp. (mainly *F. plautii*), *Alistipes* spp. (mainly *A. finegoldii* and *A. shahii*), *Ruminococcus* spp. and *Fusobacterium* spp.	• over a half of the species were significantly altered in ESRD patients, suggesting ESRD strongly affects the microbiome	(120)
	• depleted species included *Prevotella* spp. (mainly *P. copri*), *Clostridium* spp. and several butyrate producers (*Roseburia* spp., *Faecalibacterium prausnitzii* and *Eubacterium rectale*)	• the enrichment of uremic toxins in patients with ESRD is associated with gut microbiome-mediated aromatic amino acids degradation and microbial secondary bile acids biosynthesis	

ESRD patients	• elevation of *Alteromonadaceae*, *Clostridiaceae*, *Dermabacteraceae*, *Enterobacteriaceae*, *Halomonadaceae*, *Polyangiaceae*, *Moraxellaceae*, *Methylococcaceae*, *Micrococcaceae*, *Cellulomonadaceae*, *Pseudomonadaceae*, *Xanthomonadaceae*, *Verrucomicrobiaceae*	• patients with ESRD exhibited significant expansion of bacteria possessing urease, uricase, and indole- and *p*-cresol-forming enzymes and reduced bacterial families possessing butyrate-forming enzymes	(9)
	• reduction of *Lactobacillaceae*, and *Prevotellaceae.*		

ESRD patients	• four decreased (*Prevotella* sp. *885*, *Weissella confuse*, *Roseburia faecis*, and *Bacteroides eggerthii*) and three elevated species (*Alloscardovia omnicolens*, *Merdibacter massiliensis*, and *Clostridium glycyrrhizinilyticum*) were altered across non-CKD, early to advanced stages	• these results implicate specific gut microorganisms as useful biomarkers for early CKD diagnosis and prognosis monitoring	(118)
	• *Cetobacterium somerae* (mild CKD), *Candidatus Stoquefichus* sp. *KLE1796* (mild CKD), *Fusobacterium mortiferum* (moderate CKD), *Bariatricus massiliensis* (moderate CKD), *Bacteroides stercorirosoris* (moderate CKD), and *Merdimonas faecis* (advanced CKD) were altered only in particular stages		

CKD stage 4–5 patients	• elevation of *Proteobacteria*, *Enterobacteriaceae*, *Corynebacteriaceae*, *Enterococcus*	• CKD patients have increased plasma TMAO levels due to contributions from impaired renal functions and gut microbiota dysbiosis	(8)
	• reduction of *Ruminococcaceae*, *Prevotella, Roseburia, Coprococcus*		

CKD stage 2–4 cats	• reduction of *Holdemania*, *Adlercreutzia*, *Eubacterium*, *Slackia*, and *Mogibacterium*	• decreased fecal microbiome diversity and richness are associated with CKD in cats	(121)

a HD, hemodialysis; ESRD, end stage renal disease; CKD, chronic kidney disease; TMAO, trimethylamine-N-oxide.

Ren et al. investigated differences in the microbial structure based on different CKD stages. Linear discriminant analysis (LDA) indicated that *Tenericutes* and *Mollicutes* were enhanced in CKD stages 1–2, *Parasutterella* was enriched in CKD stages 3–4, and *Akkermansia*, *Blautia*, and *Verrucomicrobia* were augmented in CKD stage 5.^46^*Alphaproteobacteria*, *Streptococcaceae*, and *Streptococcus* were more abundant in adults receiving hemodialysis or peritoneal dialysis than in controls.^44^ Guirong et al. evaluated the gut microbiota composition of kidney transplant (KT) recipients reporting that their gut microbial profiles were similar to patients with CKD stages 3–4, with increased *Bacteroidetes, Proteobacteria, Clostridiales*, and *Enterobacteriaceae* but decreased *Firmicutes*, *Lachnospiraceae*, *Ruminococcaceae*, and *Faecalibacterium* compared to healthy controls.^47^

These differences in bacterial phyla and genera between patients with CKD and healthy controls suggest that the alterations in bacterial taxa may offer valuable insights into predicting CKD progression. *Akkermansia* (the area under the receiver operating characteristic curve (AUC) = 0.753),^48^*Lactobacillus* (AUC = 0.792),^48^*Ruminococcus* (AUC = 0.771),^49^ and *Roseburia* (AUC = 0.803)^49^ differentiated adults with CKD from controls. Furthermore, *Escherichia-Shigella* and *Prevotella* 9 (AUC = 0.86) could be used to accurately distinguish patients with diabetic nephropathy from age/gender-matched diabetes mellitus.^50^

### 2.3 Gut Microbial Dysbiosis Affects CKD Progression

The alteration of gut microbial profiles significantly affects CKD progression through the following mechanisms:

#### 2.3.1 Bacterial Families Possessing the Enzymes to Synthesize Uremic Toxins

Three bacterial families (*Clostridiaceae*, *Enterobacteriaceae*, and *Verrucomicrobiaceae*) contain the tryptophanase gene to produce indole. Five families (*Cellulomonadaceae*, *Dermabacteraceaea*, *Micrococcaceae*, *Polyangiaceae*, and *Xanthomonadaceae*) possess the uricase gene, and two (*Clostriadiaceae* and *Enterobacteriaceae*) can deaminate tyrosine into *p*-cresol.^9^ These bacterial families are dominant in ESRD patients. Gryp et al. reported that the gut microbiome of patients with CKD was dominated by GDUT-precursor producers including *Actinomycetaceae*, *Bacteroidaceae*, *Clostridiaceae*, *Enterococcaceae*, *Lachnospiraceae*, *Staphylococcaceae*, *Tannerellaceae*, *Bacteroides uniformis*, *Odoribacter splanchnicus*, and *Oscillibacter* sp., generating *p*-cresol, phenol, and IAA.^51^ Other families, such as *Bifidobacteriaceae*, *Coriobacteriaceae*, *Enterobacteriaceae*, *Propionibacteriaceae*, and *Rikenellaceae*, are involved in indole production.^51^ A high abundance of bacterial families with urease, uricase, and indole and *p*-cresol-forming enzymes could accelerate CKD progression by affecting the synthesis of uremic toxins.^9^

#### 2.3.2 Bacterial Families Possessing the Ability to Synthesize SCFA

*Lactobacillaceae* and *Prevotellaceae* are butyrate-producing bacteria with phosphotransbutyrylase and butyrate kinase activity, which are reduced in ESRD patients.^9^ Butyrate exerts the most biological activity in SCFA and stimulates mucin synthesis to protect intestinal homeostasis and intact antibacterial barrier.^52^ Integration of the intestinal epithelium is crucial to prevent the systemic translocation of microbial toxins and pathogens into the systemic circulation with widespread damage throughout the body.^53^ Gryp et al. revealed a lower abundance of SCFA-producing bacteria, *Bifidobacterium* spp., and *Streptococcus* spp. in patients with CKD.^51^ Similarly, Wang et al. reported a decrease in SCFA-producing bacteria in the CKD group, including *Ruminococcaceae*, *Eubacteriaceae*, *Oscillospiraceae*, *Lachnospiraceae*, *Clostridiaceae*, *Oscillospiraceae*, *Veillonellaceae*, *Rikenellaceae*, *Bifidobacteriaceae*, and *Lactobacillaceae*.^7^

#### 2.3.3 Bacterial Families Possessing/Producing Lipopolysaccharide (LPS)

LPS constitutes the outer membranes of most Gram-negative bacteria.^54^ Gram-negative bacterial families, such as *Corynebacteriaceae*, *Pseudomonadaceae*, and *Enterobacteriaceae*, are enriched in the CKD population.^8,9^ LPS activates the NF-κB pathway and mTOR signaling in macrophages to stimulate the production of proinflammatory cytokines (IL-β1, TGF-β1, MCP-1, and TNF-α), leading to the CKD progression with kidney inflammatory injuries and fibrosis.^55,56^

### 2.4 The Causal Relationship Between CKD and Gut Dysbiosis Remains Unclear

Clinical studies have highlighted microbial composition and its bidirectional relationship with CKD, contributing to disease progression.^57^ However, the causality between CKD progression and gut dysbiosis has not been fully elucidated; therefore, further exploration of the causal relationship between gut dysbiosis and CKD is required to clarify the potential pathogenesis of CKD progression. Xu et al. transferred fecal microbiota from patients with CKD and healthy controls into antibiotic-treated C57BL/6 mice, showing that the CKD fecal samples resulted in significantly higher plasma TMAO levels in mice with increased *Clostridium* and *Parabacteroides*, along with decreased *Ruminococcaceae* and *Megamonas* compared to the group that received healthy fecal microbes.^8^ Wang et al. revealed that ESRD-specific gut microbiota induced systemic inflammation and colonic epithelial barrier defects in germ-free rats with a significant increase in fecal phenol and phenol-producing bacteria (*Bacteroides* and *Escherichia*), suggesting that gut microbiota from ESRD patients led to gut barrier defects by excessive phenol production.^58^

Until now, manipulating gut microbiota via fecal microbiota transplantation (FMT) from patients with ESRD or CKD into germ-free animals has not provided convincing evidence to illustrate the detrimental effect on renal function. The parameters of FMT operation, including frequency, dosage of fecal microbiota, and duration, should be considered in future investigations to establish the causative relationship between gut dysbiosis and CKD. However, several studies demonstrated that the gut microbiota significantly aggravate or alleviate CKD progression. Fecal microbiota from CKD rats transplanted into 5/6 nephrectomy rats increased protein-bound uremic toxins and a decline in renal function compared to the nontransplanted 5/6 nephrectomy rats. However, these adverse effects were significantly mitigated when fecal microbiota from healthy recipients was transplanted.^59^ Similarly, CKD mice that underwent FMT from healthy mice demonstrated a noticeable improvement in gut microbiota disturbance and a reduction in PCS accumulation.^60^ Collectively, these studies showed that manipulating the gut microbiota in CKD is a potential approach to alleviate disease progression.

## 3 Alleviating Effects of Probiotics on Ckd via the Gut–Kidney Axis

The strong linkage between CKD and gut microbial dysbiosis suggests that modifying the gut microbiota could potentially diminish uremic toxin levels and associated adverse effects. Probiotics, defined as “live microorganisms that, when administered in adequate amounts, exist a health benefit on the host”^10^ exert a positive impact on CKD alleviation by attenuating gut microbiome disturbances.

### 3.1 *In Vitro* Preselection Platforms with *in Vivo* Verification are Feasible Strategies for Potential CKD-Alleviating Probiotic Screening

Preselection of suitable probiotic strains based on their beneficial attributes is crucial because their physiological functions are highly strain-specific.^61^ However, most studies have not elucidated the probiotic selection in detail, so the development of an *in vitro* screening platform is essential for providing an opportunity to mass-screen potential microorganisms.^11^ A previous study created a probiotic screening platform based on gut-derived uremic toxin-reducing probiotics and successfully preselected three probiotic strains, *Lactobacillus paracasei* subsp. *paracasei* BCRC 12188, *Streptococcus salivarius* subsp. *thermophiles* BCRC 13869, and *Lactobacillus plantarum* subsp. *plantarum* BCRC 12251 due to their ability to reduce IS *in vitro*.^12^*In vivo*, oral administration of a combination of the three strains (Pm1) significantly suppressed IS accumulation in the serum, kidneys, and liver in a cisplatin-induced acute kidney injury mouse model.^12^ A cisplatin-induced minipig model also demonstrated a lower incidence of lesions, including atrophy, mononuclear inflammation, cell infiltration, and interstitial fibrosis in renal tubules in the high-dosage Pm1 group.^62^ A significant reduction of blood urea nitrogen (BUN) and creatinine (CRE) was also observed compared with the cisplatin group. The possible mechanism involves the downregulation of inflammatory cytokine production and the upregulation of plasma superoxide dismutase activity. The modulation of gut microbiota was also observed after Pm1 intervention, with a decreasing abundance of Gram-negative bacteria contributing to reduced inflammation and apoptosis in the kidney, preventing CKD progression.^62^

A recent study preselected two potential strains (*Lactobacillus plantarum* subsp. *plantarum* MFM 30–3 and *Lactobacillus paracasei* subsp. *paracasei* MFM 18) using a modified screening platform to determine the ability to remove uremic toxin precursors in simulated intestinal juice.^11^ Furthermore, an *in vivo* study in an adenine-induced renal injury mouse model demonstrated that the renal dysfunction features, including high levels of BUN, CRE, IS, and PCS in plasma, interstitial fibrosis, and kidney injuries, were reduced by intervention with the preselected probiotics, which was accompanied by improvement of gut dysbiosis and prevention of intestinal barrier disruption via modulation of metabolite production.^11^

Developing an *in vitro* screening platform to identify strains that can reduce uremic toxin levels with further *in vivo* verification is a feasible strategy for potential CKD-alleviating probiotic screening. However, the speed of removal of a toxic compound is strain-specific and affected by the physiological state of the strain, pH, and nutrients.^63^ Other selection criteria, including decreasing indole production by *Escherichia coli*,^64^ antipathogenic,^64,65^ antioxidant,^66,67^ anti-inflammatory,^68,69^ and gut barrier protection^70,71^ are highly recommended to increase the therapeutic potentials for CKD.

### 3.2 The Positive Effects on CKD Alleviation after Probiotics Intervention

Table 3 summarizes the clinical trials examining the use of probiotics in patients with CKD. A total of 12 trials each were conducted for both dialysis patients and nondialyzed patients with stage 2–5 CKD. One trial was conducted on kidney transplantation patients. The sample size ranged between 9 and 70 patients, and the study duration varied from 2 to 24 weeks. Probiotic agent dosages ranged from 1.1 × 10^7^ to 2.0 × 10^12^ CFU. The probiotic agents were in various forms, including sachets, powders, and capsule.

**Table 3:** Summary of Clinical Trials Examining the Use of Probiotics in Patients with Chronic Kidney Disease^a^

study design/intervention duration/country	probiotics	dosage	main findings	ref
a retrospective study	*Lactobacillus* (*Lactiplantibacillus*) *plantarum* MFM30-3	1 × 10^10^ CFU/day	• reduction of creatinine	(77)
kidney transplatation patients (*n* = 24)	*Lactobacilus* (*Lacticaseibacillus*) *paracasei* MFM 18		• improvement of eGFR	
3 months				
Taiwan				

a single-arm pilot study	• powder sachet (2 g):	2 sachets/day	• fecal SCFAs increased significantly	(73)
hemodialysis patients (*n* = 18)	*Bifidobacterium bifidum* BGN4: 7 × 10^9^ CFU		• reduction of serum calprotectin, a marker of acute inflammation, decreased significantly	
3 months	*Bifidobacterium longum* BORI: 2 × 10^9^ CFU		• serum TNF-α and IL-6 upon LPS stimulation significantly decreased	
Korea			• reduced systemic inflammatory responses were associated with an increase in Tregs and a decrease in proinflammatory monocytes	

a randomized, single-blind, placebo-controlled pilot trial	• synbiotic bag:	2 bags/day	• reduction of serum free IS	(76)
CKD stage 3b-4 patients (*n* = 47)	*Lactobacillus* (*Lacticaseibacillus*) *casei* LC4P1: 2.4 × 10^9^		• reduction of small intestinal permeability	
2 months	*Bifidobacterium animalis* BLC1: 2.4 × 10^9^		• amelioration of abdominal pain and constipation syndrome	
Italy	fructooligosaccharides 2.5 g			
	inulin 2.5 g			
	natural antioxidants (quercetin 0.064 g, resveratrol 0.023 g, and proanthocyanidins 0.013 g)			

a randomized, double-blinded, placebo-controlled clinical trial	*Lactobacillus acidophilus*	2 × 10^12^ CFU/day	• improvement of gastrointestinal symptoms	(98)
hemodialysis patients (*n* = 18)	*Bifidobacterium bifidum*	2.31 g/day	• elevation of *Bifidobacterium*	
2 months	inulin			
Mexico				

a double-blind, randomized clinical trial	*Lactobacillus* (*Lactiplantibacillus*) *plantarum A87*	4 × 10^10^ CFU/day	• reduction of serum CRP, glucose, and syndecan-1 (endothelial lesion marker)	(100)
hemodialysis patients (*n* = 70)	*Lactobacillus* (*Lacticaseibacillus*) *rhamnosus*			
3 months	*Bifidobacterium bifidum A218*			
Brazil	*Bifidobacterium longum A101*			

a double-blind, randomized, placebo-controlled clinical trial	*Lactobacilus* (*Lacticaseibacillus*) *casei*	1 g/day	• reduction of BUN and trend to decrease serum CRE	(78)
CKD stage 3–4 patients (eGF*R* = 15–59 mL/min per 1.73 m^2^) (*n* = 66)	*Lactobacilus acidophilus*			
6 weeks	*Lactobacilus bulgarigus*			
Iran	*Lactobacilus* (*Lacticaseibacillus*) *rhamnosus*			
	*Bifidobacterium breve*			
	*Bifidobacterium longum*			
	*Sterptococus thermophilus*			
	Fructooligosaccharides			

a double-blind, randomized, placebo-controlled clinical trial	*Lactobacillus* (*Lacticaseibacillus*) *rhamnosus*	1.6 × 10^7^ CFU/day	• reduction of serum *p*-cresol and phenol	(79)
hemodialysis patients (*n* = 42)				
4 weeks				
Iran				

a double-blind, randomized, placebo-controlled trial	• powder packets (5 g):	3 packets/day	• reduction of plasma *p*-cresol	(80)
nondialyzed CKD stage 3–4 patients (eGF*R* = 15–60 mL/min per 1.73 m^2^) (*n* = 30)	*Lactobacillus* (*Lactiplantibacillus*) *plantarum*: 5 × 10^10^			
4 weeks	*Lactobacillus* (*Lacticaseibacillus*) *casei* subsp. *rhamnosus*: 2 × 10^9^			
Italy	*Lactobacillus gasseri*: 2 × 10^9^			
	*Bifidobacterium infantis*: 1 × 10^9^			
	*Bifidobacterium longum*: 1 × 10^9^			
	*Lactobacillus acidophilus*: 1 × 10^9^			
	*Lactobacillus* (*Ligilactobacillus*) *salivarius*: 1 × 10^9^			
	*Lactobacillus sporogenes*: 1 × 10^9^			
	*Streptococcus thermophiles*: 5 × 10^9^			
	inulin: 2.2 g			
	tapioca-resistant starch:1.3 g			

a single-arm pilot study	*Bifidobacterium infantis*	6 × 10^8^ CFU/day	reduction of fecal indole and *p*-cresol, and plasma indican	(81)
hemodialysis patients (*n* = 20)	*Lactobacillus acidophilus*			
4 weeks	*Enterococcus faecalis*			
Japan	the ratio of probiotic strain: 1:1:1			

a prospective, single-arm study	*Lactobacillus acidophilus* La-14	1.5 × 10^10^ 65 mg	• reduction of serum PCS, IS, IL-6, and MDA	(74)
hemodialysis patients (*n* = 30)	fructooligosaccharides			
8 weeks				
Bulgaria				

a randomized, double-blinded, placebo-controlled clinical trial	*Lactococcus lactis* subsp. *lactis* LL358	2 sachets/day	• reduction of serum IS	(82)
hemodialysis patients (*n* = 50)	*Lactobaccillus* (*Ligilactobacillus*) *salivarius* LS159	1 × 10^11^ CFU/day		
6 months	*Lactobaccillus (Lactiplantibacillus) pentosus* LPE588			
Taiwan				

a randomized, double-blinded, placebo-controlled clinical trial	*Bifidobacterium longum* NQ1501	*B. longum*	• probiotics did not significantly alter species diversity (Chao1 and Shannon) of the fecal microbiome, but induced a rearrangement in the microbial composition	(83)
hemodialysis patients (*n* = 45)	*Lactobacillus acidophilus* YIT2004	3.7 × 10^9^ CFU/day		
6 months	*Enterococcus faecalis* YIT0072	*L. acidophilus*		
China		8.9 × 10^8^ CFU/day	• probiotics reduced serum indole-3-acetic acid-*O*-glucuronide, *m*-cresol, *p*-cresol, and phenol of nondiabetic patients	
		*E. faecalis*		
		1.8 × 10^9^ CFU/day		

a simple randomized, controlled clinical trial	*Lactobacillus* (*Lacticaseibacillus*) *casei* shirota 8 × 10^9^ CFU in fermented dairy drink	8 × 10^9^ CFU/day	• reduction of blood urea in 16 × 10^9^ CFU group	(84)
CKD stage 3–4 patients (eGF*R* = 15–59 mL/min per 1.73 m^2^) (*n* = 30)		16 × 10^9^ CFU/day		
2 months				
Mexico				

a double-blind, randomized, placebo-controlled trial	• synbiotic pill:	2 pills/day	• reduction of serum IS and trend to decrease serum PCS and TMAO	(14)
nondialyzed CKD patients (eGF*R* = 15 −45 mL/min per 1.73 m^2^) (*n* = 34)	*Lactobacillus acidophilus* CBT LA1: 4 × 10^9^		• improvement of eGFR	
12 weeks	*Lactobacillus* (*Lacticaseibacillus*) *casei* CBT LC5: 4 × 10^9^		• decreased level of high-sensitivity CRP	
Serbia	*Bifidobacterium lactis* CBT BL3: 8 × 10^9^		• enrichment of *Bifidobacteria*, *Lactobacillus*, and *Subdoligranulum*	
	inulin: 1.6 g			

a preliminary study	*Lactobacillus* (*Lacticaseibacillus*) *casei* Shirota (LcS)	3 × 10^8^ CFU/day	• reduction of serum *p*-cresol	(85)
hemodialysis patients (*n* = 9)	*Bifidobacterium breve* Yakult (BbY)	5.01 g	• improvement of difficulty in defecation and increased stool quantity	
2 weeks	galacto-oligosaccharides (GOS)			
Japan				

a double-blind, randomized, placebo-controlled crossover study	*Streptococus thermophilus* KB19	1.8 × 10^12^ CFU/day	• the trend of reductions of serum CRP and total indoxyl glucuronide	(86)
hemodialysis patients (*n* = 22)	*Lactobacillus acidophilus* KB27			
2 months	*Bifidobacterium longum* KB31			
USA				

a pilot-scale, randomized, double-blind, placebo-controlled trial	*Lactobacillus acidophilus* KB31	9 × 10^10^ CFU/day	• reduction of serum BUN and uric acid	(87)
CKD stage 3–4 patients (*n* = 13)	*Bifidobacterium longum* KB35		• elevation of *Lactobacillus* and *Streptococcus*	
3 months	*Streptococus thermophilus* KB27		• reduction of fecal pH	
Canada				

a prospective, randomized, double-blind, placebo-controlled, crossover trial at five institutions	*Streptococus thermophilus* KB19	9 × 10^10^ CFU/day	• reduction of BUN and trend to decrease serum CRE and uric acid	(88)
CKD stage 3–4 patients (*n* = 46)	*Lactobacillus acidophilus* KB27		• improvement of quality of life	
3 months	*Bifidobacterium longum* KB31			
USA, Canada, Nigeria, and Argentina				

a double-blinded, placebo-controlled, randomized clinical trial	*Lactobacillus* genera	9 × 10^9^ CFU/day	• reduction of serum PCS and trend to decrease serum IS	(89)
nondialyzed CKD stage 4–5 patients (eGF*R* = 10–30 mL/min per 1.73 m^2^) (*n* = 31)	*Bifidobacteria* genera	15 g	• elevation of *Bifidobacterium* and trend to increase *Lactobacillus*	
6 weeks	*Streptococcus* genera			
Australia	with nine different strains			
	inulin			
	fructo-oligosaccharides			
	galacto-oligosaccharides (GOSs)			

a single-center, open-label, randomized, placebo-controlled study	• Enterelle capsule (0.377 g):	• week 1: Enterelle ×3/day	• probiotics-treated patients exhibited a significant reduction of urinary indican and 3-methyl-indole.	(90)
CKD stage 3a patients (eGFR = 45–60 mL/min per 1.73 m^2^) (*n* = 28)	*Enterococcus faecium* (UBEF-41)	• week 2–3 Bifiselle ×3/day	*• Lactobacillales* and bifidobacteria concentrations were increased in the probiotics group	
15 weeks		Ramnoselle ×3/day		
Italy	*Lactobacillus acidophilus* (LA-14) *Saccharomyces cerevisiae* subsp. *Boulardii* (MTCC-5375)	• Week 4–15:		
	• bifidobacteria capsule (0.455g):	Bifiselle ×2/day		
	*Bifidobacterium brevis* (BB03)	Ramnoselle ×2/day		
	*Bifidobacterium bifidum* (BB06)			
	*Bifidobacterium longum* (BL05)			
	• Ramnoselle capsule (0.455 g):			
	*Lactobacillus* (*Lacticaseibacillus*) *rhamnosus* (HN-001)			
	*Lactobacillus* (*Lacticaseibacillus*) *rhamnosus* (LR-32)			
	*Lactobacillus acidophilus* (LA-14)			

• a preliminary study	*Bifidobacterium longum*	3 × 10^9^ CFU/day	reduction of serum IS	(91)
hemodialysis patients (*n* = 11)				
5 weeks				
Japan				

a double-blinded, placebo-controlled, randomized clinical trial	*Lactobacillus acidophilus NCFM*	11 × 10^6^ CFU/day	• improvement of gastrointestinal symptoms	(99)
hemodialysis patients (*n* = 42)	*Bifidobacterium lactis Bi-07*	2.31 g/day	• the trend to decrease CRP	
2 months	inulin			
Mexico				

a randomized, double-blinded, placebo-controlled trial	*Bifidobacterium bifidum A218*	4 × 10^9^ CFU/day	• reduction of serum endotoxin and proinflammatory cytokine (TNF-α, IL-6, and IL-5)	(75)
peritoneal dialysis patients (*n* = 39)	*Bifidobacterium catenulatum A302*		• elevation of anti-inflammatory cytokine (IL-10)	
6 months	*Bifidobacterium longum A101*			
Taiwan	*Lactobacillus* (*Lactiplantibacillus*) *plantarum A87*			
	the ratio of probiotic strain: 1:1:1:1			

a single-arm pilot study	*Lactobacillus acidophilus* TYCA06	1 × 10^10^ CFU/day	• eGFR decline was significantly slower	(64)
nondialyzed CKD stage 3–5 patients (*n* = 38)	*Bifidobacterium longum* subsp. *infantis* BLI-02		• serum TNF-α, IL-6, IL-18, and endotoxin were significantly decreased	
6 months	*Bifidobacterium bifidum* VDD088		• improvement of gastrointestinal symptoms (borborygmus and flatulence)	
Taiwan	the ratio of probiotic strain:1:1:1		• the abundance of *B. bifidum* and *B. breve* increased significantly	

a randomized, double-blinded, placebo-controlled clinical trial	*Lactobacillus* (*Lacticaseibacillus*) *casei Zhang*	4 × 10^9^ CFU/day	• log10 urine albumin-to-creatinine ratio (UACR) mildly increased in the probiotic group compared to a significant increase in the placebo group	(92)
nondialyzed CKD stage 3–5 patients (*n* = 62)			• the amplitude of the increase in CRE was lower in the probiotic group after 1 year observation	
3 months			• eGFR decline was slower in the probiotic group during the intervention and follow-up 7 months	
China			• the diversity of intestinal flora did not change among groups	

a CFU, colony-forming unit; CKD, chronic kidney disease; CRE, creatinine; BUN, blood urea nitrogen; eGFR, estimated glomerular filtration rates; IS, indoxyl sulfate; PCS, *P*-cresyl sulfate; TMAO, trimethylamine-*N*-oxide; SCFA, short-chain fatty acid; CRP, C-reactive protein; TNF-α, tumor necrosis factor-α; IL-5, interleukin-5; IL-6, interleukin-6; IL-10, interleukin-10; IL-18, interleukin-18; MDA, malondialdehyde.

The various probiotic strains evaluated were within nine genera (*Bifidobacterium*, *Enterococcus*, *Lacticaseibacillus*, *Lactiplantibacillus*, *Lactobacillus*, *Lactococcus, Ligilactobacillus*, *Saccharomyces*, and *Streptococcus*) according to new *Lactobacillus* taxonomy.^72^ These probiotic species have been proven to function in many pathological and physiological processes, including the regulation of immune capacity,^64,73−75^ alleviation of oxidative stress,^74^ protection of the gastrointestinal tract,^76^ reduction of plasma BUN, CRE, and renal toxins.^74,76−92^ It is worth noting that intervention with multiple probiotic strains in human trials is preferred (20 of 25 studies) due to a broader range of health-promoting effects and synergistic mechanisms of action,^93^ suggesting a higher opportunity for success.^94^ However, insufficient evidence supports a better health-promoting effect when using multistrain probiotics than single-strain probiotics.^95^ Therefore, the selection of probiotic strains for human trials must be based on *in vitro* and *in vivo* scientific evidence.

Most studies reported positive effects such as reduced uremic toxins (IS, PCS, TMAO, indoxyl glucuronide) or precursors of uremic toxin (indole, *p*-cresol, and phenol) after the probiotic intervention.^14,74,76,79−83,85,86,89−91^ However, only seven trials^14,64,77,78,87,88,92^ observed an improved renal function index, including reduced BUN and CRE, and slowing eGFR decline. The primary function of specific probiotics is an adjuvant strategy used to restore microbial balance and suppress circulating levels of uremic toxins,^96^ so improved renal function indicators may not be observed at this stage. Additionally, BUN and CRE levels are influenced by dietary and physiologic conditions unrelated to renal function,^97^ which might affect the statistical significance of clinical studies. Besides renal function, some studies also observed improved gastrointestinal symptoms (borborygmus, flatulence, abdominal pain, and constipation syndrome),^64,76,85,98,99^ gut microbial modulation (upregulation of lactobacilli and bifidobacteria),^14,64,87,89,90,98^ and anti-inflammatory effects.^64,73−75,14,86,99,100^ Inflammatory biomarkers were evaluated in seven studies, with decreased inflammatory cytokines (TNF-α, IL-5, IL-6, IL-18), CRP, and LPS in serum compared to controls after probiotics intervention.

Synbiotic supplementation also positively affected renal function and gut microbiota in patients with CKD.^14,78,89,98^ Synbiotic therapy significantly reduced serum IS and PCS in patients with CKD, with the enrichment of *Bifidobacterium* and *Lactobacillus* and depletion of *Ruminococcaceae*. Moreover, a significant negative correlation was observed between changes in the relative abundance of *Bifidobacterium* and serum PCS and IS concentrations.^89^

Attempts to restore the desired microbiome by introducing favorable microorganisms seem feasible for CKD alleviation. However, the limited size of the study cohorts and the relatively short follow-up period hinder a comprehensive understanding of probiotics’ efficacy in treating individuals with CKD. Therefore, further long-term basic and clinical studies are needed in the future.

### 3.3 The Gut–Kidney Axis Plays a Vital Role in the Preventive/Therapeutic Mechanisms of Probiotics in CKD

The probiotics’ potential efficacy in managing CKD is through the gut–kidney axis by modulating gut microbial composition and improving gut dysbiosis, which further reduces uremic toxins, increases SCFA, enhances gut barrier integrity, ameliorates gastrointestinal symptoms, and decreases the inflammatory response, contributing to alleviating CKD progression.

#### 3.3.1 Modulating Gut Microbial Composition and Improving Gut Dysbiosis

Numerous studies reveal that orally administered probiotics in animal models slow the progression of kidney disease by correcting the intestinal microbial imbalance.^11,13,101^ Six clinical studies analyzed gut microbiota after probiotics intervention in patients with CKD using high throughput sequencing, and the results support the importance of the gut–kidney axis in alleviating CKD.^14,64,73,83,89,92^ In a single-center double-blind, randomized, placebo-controlled study, administration of Bifco capsules, which is a mixture of viable bacteria (*Enterococcus faecalis*, *Bifdobacterium longum*, and *Lactobacillus acidophilu*s), for six months induced microbial composition changes and reduced the relative abundance of *Ruminococcaceae*, *Halomonadaceae*, *Peptostreptococcaceae*, and *Erysipelotrichacease* and elevated *Bacteroidaceae* and *Enterococcaceae* in nondiabetic hemodialysis patients.^83^ In another 6-month single-arm pilot study, the dominant genera of the intestinal microbiome changed during the probiotic intervention.^64^ In a double-blind, randomized, placebo-controlled trial, intervention with synbiotic pills (*Lactobacillus acidophilus* CBT LA1, *Lacticaseibacillus casei* CBT LC5, *Bifidobacterium lactis* CBT BL3, and inulin), the relative abundance of *Bifidobacterium*, *Lactobacillus*, and *Subdoligranulum* significantly increased compared to the placebo group.^14^ In a small-scale study with a probiotics intervention (mixture of *Bifidobacterium bifidum* BGN4 and *Bifidobacterium longum* BORI) for three months, the population of *Prevotella*, *Enterococcus*, *Alistipes*, *Clostridia*, *Escherichia-Shigella*, *Klebsiella*, and *Bifidobacterium* increased while *Bacteroides*, *Faecalibacterium*, *Eubacterium siraeum*, *Tyzzerella*, *Sutterella*, and *Akkermansia* reduced.^73^

Through analyzing the microbial composition based on the reviewed clinical studies, the CKD alleviating effect of probiotics on modulating gut microbiota involves:

##### 3.3.1.1 Upregulating the Beneficial Bacterial Genus *Bifidobacterium*

Among the clinical trials, six studies noted significant increases in *Bifidobacterium,* which parallels other fecal microbial analyses in patients with CKD using culture-dependent, qPCR quantification or next-generation sequencing approaches.^14,64,73,89,90,98^*Bifidobacterium*, decreased in patients with CKD,^45^ has been reported to promote colon health by increasing the production of microbial metabolites, such as SCFA,^102^ and the slow CKD progression positively relates to the eGFR improvement.^14,51^

##### 3.3.1.2 Upregulating Bacterial Families Possessing the Enzymes to Synthesize SCFA

Other bacterial genera possessing enzymes to synthesize SCFA are enriched after probiotic intervention, such as *Lactobacillus* and *Subdoligranulum*.^14,103^ These bacteria convert dietary fiber in the gut into monosaccharides through a series of reactions mediated by specific enzymes.^104^ A reduction in *Bifidobacterium*, *Lactobacillus*, and SCFA producers, such as *Prevotella* spp., *Clostridium* spp., *Roseburia* spp., *Enbacterium* spp., *Coprococcus* spp., and *Faecalibacterium prausnitzii* have been observed in the patients with CKD.^9,45,105−107^ Furthermore, SCFA producers (*Butyricicoccus* spp., *F. prausnitzii*, *Roseburia* spp., and *Bifidobacterium* spp.) showed an inverse correlation with the severity of CKD progression and increased with higher eGFR.^51^ Additionally, SCFAs produced by gut bacteria and delivered to the kidney through the peripheral circulation could protect tubular cells from oxidative cellular stress, mitigate renal ischemia-reperfusion injury, reduce inflammation, lower the infiltration of immune cells, and diminish apoptotic cells in injured kidneys.^108^ Thus, increased SCFA-producing bacteria in the gut after probiotic intervention could improve gut health and positively affect CKD.^109,110^

##### 3.3.1.3 Downregulating the Bacterial Families Possessing the Enzymes to Synthesize Uremic Toxins

A high abundance of gut bacterial families with enzymes to synthesize uremic toxins could accelerate CKD progression. Indole, phenol, and *p*-cresol are aromatic compounds produced by intestinal bacteria via aromatic amino acids (tryptophan and tyrosine).^9,51,110^ Upregulating nonphenol-producing bacteria, such as *Subdoligranulum*, genera of the *Ruminococcaceae* family,^14^ and downregulating indole-producing bacteria, such as *Escherichia* spp.,^64^ were observed in patients with CKD after the probiotic intervention. Reducing the *Ruminococcaceae* family was also reported in HD patients supplemented with probiotics.^83^ Some bacteria from *Ruminococcaceae* can ferment tyrosine to *p*-cresol,^110^ and modulation of *Ruminococcaceae* appears to correlate with a healthier gut environment in CKD patients.

##### 3.3.1.4 Downregulating the Bacterial Families Associated with Inflammation

Reduction of endotoxin-producing Gram-negative bacteria, such as *Megamonas*, *Escherichia-Shigella*, and *Halomonadaceae*, in patients with CKD with probiotic intervention indicates decreasing chronic immune responses associated with inflammation in the human gut. Both studies show a reduction in the serum levels of endotoxin.^64,75,83^ Several reports found more *Halomonadaceae* in ESRD patients.^9,111^ The observed decline in these bacteria suggests that probiotics may potentially restrain their overgrowth in CKD patients.

#### 3.3.2 Reducing Serum Uremic Toxins/Endotoxin and Increasing Fecal SCFA via Modulating Gut Microbiota

Probiotic intervention modulates gut microbial composition, thereby down-regulating the bacterial families that synthesize uremic toxins. Reduced precursors of uremic toxins (indole, *p*-cresol, and phenol)^79−81,83,85,90^ or uremic toxins (IS, PCS, TMAO, indoxyl glucuronide)^14,74,76,81−83,86,89−91^ were observed in 13 trials of patients with CKD after probiotic intervention. Probiotic intervention also reduces the levels of endotoxin/LPS by inhibiting Gram-negative bacteria. LPS, a major component of the outer membrane in Gram-negative bacteria.^112^ Serum endotoxin levels were significantly decreased in patients with CKD receiving probiotics.^64,75,83^ Additionally, the probiotic intervention also upregulates bacterial families possessing the enzymes to synthesize SCFA.^73^

#### 3.3.3 Reducing Inflammation, Oxidative Stress, and Intestinal Barrier Injury via Reducing Serum Uremic Toxins/Endotoxin and Increasing SCFAs

SCFAs produced by the intestinal microbiome can reduce inflammation and oxidative stress. The mechanisms involved in the anti-inflammatory response include decreasing the proliferation of immune cells and cytokine levels, inhibiting NF-κB, reducing neutrophil recruitment,^113^ and a direct effect on T cells through binding to specific receptors (GPR41, GPR43, and GPR109A).^104^ SCFAs can modulate the Keap1-Nrf2-dependent cellular signaling pathway to maintain redox homeostasis.^114,115^ Detrimental effects of GDUTs, including oxidative stress, inflammation, fibrosis, kidney failure, and insulin resistance, have been well documented,. Thus, by downregulating GDUTs and upregulating SCFAs, probiotics reduce inflammation, oxidative stress, and intestinal barrier injury in patients with CKD. Probiotics have been shown to decrease serum proinflammatory cytokines,^64,73,75^ increase T regulatory cytokines,^75^ reduce small intestinal permeability,^76^ increase fecal SCFAs,^73^ and reduce serum GDUTs^14,76,81−83,86,89−91^ and endotoxins^64,75^ in patients with CKD. These findings verify that the gut–kidney axis significantly impacts the preventive/therapeutic mechanisms of probiotics in CKD.

## 4 Further Considerations and Concluding Remarks

Gut dysbiosis contributes to deteriorating CKD progression; thus, probiotics are a potential strategy to restore the desired microbiome and treat CKD. Clinical studies have revealed the various physiological functions of probiotics in patients with CKD including reduction of uremic toxins and related precursors, modulation of gut microbiota, regulation of immune capacity, protection of the gastrointestinal tract, and improvement of gastrointestinal symptoms. However, only approximately 25% of human cohorts have shown a positive effect on renal function, which is the most critical outcome demonstrating the efficacy of probiotics in improving CKD. The considerable heterogeneity in the responses to probiotic treatment in CKD may be due to individual factors such as diet, age, physiological condition, immune response, and indigenous gut microbiota,^116^ as well as differing expression of mucosal immune-related genes in gastrointestinal organs, leading to personalized colonization patterns.^117^

Nonetheless, a strong relationship exists between CKD-related specific gut microbial profiles and metabolites and their interplay with probiotics. Thus, it is necessary to investigate alterations in the bacterial communities and bacterial-related metabolic functions to select appropriate probiotics to treat CKD and understand their underlying mechanisms of action. Human clinical studies should involve the integrated analysis of microbial configurations and metabolite profiles during probiotics intervention using a combination of multiomic technologies, such as metagenomics, metabolomics, and transcriptomics, which could provide more precise insights into the mechanisms of probiotics function.

CKD severity shapes varying statuses of gut dysbiosis, indicating a descending trend of gut diversity and abundance with CKD progression.^7,118^ Clinical studies are usually conducted with specific CKD populations, which make it interesting whether disease severity influences gut modulation and subsequent probiotics effects. Such investigations could help suggest favorable timing of probiotic interventions for better outcomes. A large-scale prospective longitudinal clinical study of varying levels of impaired kidney function and a long follow-up period should be conducted to comprehensively understand probiotics’ efficacy in treating CKD. In addition, it is recommended that commensal bacteria be identified as novel microbiota-based biomarkers to monitor disease progression and facilitate the development of next-generation probiotics or precision probiotics to prevent CKD progression.

In conclusion, although several clinical studies have demonstrated the positive impacts of probiotics on various outcomes in patients with CKD, their effectiveness in CKD treatment remains a subject of debate and requires further verification. It is anticipated that future large-scale, long-term studies will confirm the potential benefits of utilizing probiotics as an adjuvant therapy in CKD.

## Abbreviations

AUC: area under curve
BUN: blood urea nitrogen
CRE: creatinine
CKD: chronic kidney disease
CVD: cardiovascular disease
eGFR: estimated glomerular filtration rate
ESRD: end-stage renal disease
FMO: flavin monooxygenase isoform
FMT: fecal microbiota transplantation
GDUT: gut-derived uremic toxin
IAA: indole-3 acetic acid
IgA: immunoglobulin A
IS: indoxyl sulfate
KDIGO: Kidney Disease Improving Global Outcomes
KT: kidney transplant
LDA: linear discriminant analysis
Lm: *Lactobacillus* mix
LPS: lipopolysaccharide
OAT: organic anion transporter
PAG: phenylacetylglutamine
PCS: *p*-cresyl sulfate
SCFA: short-chain fatty acid
TMA: trimethylamine
TMAO: trimethylamine-*N*-oxide
